# Indications for Additional Pedicle Subtraction Osteotomy in Iatrogenic Flatback After Short-Segment Fusion Surgery

**DOI:** 10.3390/medicina61091624

**Published:** 2025-09-08

**Authors:** Sung-Min Kim, In-Seok Son, Yong-Chan Kim, Xiongjie Li, Maolin Jin

**Affiliations:** Department of Orthopaedic Surgery, Kyung Hee University Hospital at Gangdong, College of Medicine, Kyung Hee University, Seoul 05278, Republic of Korea; osdrksm83@gmail.com (S.-M.K.); wiselove@naver.com (I.-S.S.); yckimspine@gmail.com (Y.-C.K.); maolinjin2@gmail.com (M.J.)

**Keywords:** iatrogenic flatback, deformity, anterior column realignment, pedicle subtraction osteotomy, sagittal balance

## Abstract

*Background and Objectives*: This study aimed to identify radiographic predictors and optimal cut-off values for determining the need for additional pedicle subtraction osteotomy (PSO) in patients with iatrogenic flatback syndrome following short-segment (≤3 levels) fusion surgery. *Materials and Methods*: From 2011 to 2022, a total of 49 patients who underwent deformity correction for iatrogenic flatback following short-segment fusion at a single institution were included. We divided all patients into group A (*n* = 33, only anterior column realignment, ACR) and group B (*n* = 16, ACR combined with PSO). Among group A patients, we further divided them into two subgroups: The Excessive group, who developed excessive anterior disc height distraction (EADH) during surgery, and the Non-excessive group, who did not. The Receiver Operating Characteristic (ROC) curve was used to determine the cut-off values for spinopelvic parameters associated with the decision to perform additional PSO. *Results*: Group A had a significantly lower number of previously fused segments compared to Group B (*p* < 0.001). Preoperative C7 sagittal vertical axis (C7SVA, *p* = 0.026) and its correction (*p* = 0.003) in group B were greater than those in group A. Group B showed a significantly more kyphotic preoperative fused segment angle (FSA) compared to Group A (*p* = 0.001). Postoperatively, EADH occurred in 7 patients (21.2%) in Group A, while no cases were observed in Group B. Subgroup analysis revealed that the dynamic segment angle (DA) was significantly lower in the Excessive group compared to the Non-excessive group (*p* < 0.001). The optimal cut-off values of preoperative radiographic parameters for selecting PSO were: C7-SVA > 242.8 mm, FSA > −3.2°, and DA < 4.3°. *Conclusions*: ACR alone and ACR combined with PSO showed satisfactory outcomes in patients with iatrogenic flat back. For selected patients with preoperative C7SVA > 242.8 mm, FSA > −3.2°, or DA < 4.3°, additional PSO may be reasonable to help optimize sagittal alignment.

## 1. Introduction

The “Iatrogenic Flatback” commonly refers to a postoperative spinal deformity characterized by a reduction in lumbar lordosis (LL) following spinal fusion surgery, leading to sagittal imbalance and back pain [1,2]. When sagittal imbalance is mild, conservative treatment can be used to manage symptoms. However, as spinal deformity advances, compensatory mechanisms gradually diminish, eventually resulting in severe sagittal imbalance that necessitates reconstructive surgery [3]. In the early era of modern spinal instrumentation, iatrogenic loss of lumbar lordosis was commonly associated with the use of straight rods and distractive techniques, particularly when applied to distal segments, resulting in “fixed” iatrogenic flatback [4]. Previous studies have shown that pedicle subtraction osteotomy (PSO) yields acceptable radiographic correction and clinical outcomes in patients with “fixed” iatrogenic flatback [3,5,6]. Furthermore, the rate of major complications is comparable to that observed in primary adult spinal deformity surgeries [7].

Nowadays, the use of pedicle screws combined with interbody cages has been shown to be effective in restoring physiological lumbar lordosis in the surgical treatment of degenerative spondylolisthesis and spinal stenosis, regardless of the presence of instability. Nevertheless, achieving the desired degree of lordosis can be challenging when cage placement or rod contouring is suboptimal. Furthermore, loss of the initially corrected angle may occur over time due to complications such as pseudarthrosis or adjacent segment degeneration, which are more frequently observed in contemporary cases of iatrogenic flatback following short-segment fusion surgery [8,9,10]. In such cases, where some unfused segments remain in the lumbar region, referred to as ‘flexible’ iatrogenic flatback, anterior column realignment (ACR) is recommended due to its advantage in achieving harmonious alignment with fewer mechanical complications [11,12]. Nonetheless, in cases of severe sagittal imbalance or thoracolumbar kyphosis requiring substantial correction angles, additional PSO may be necessary [13].

However, there is still no consensus on the optimal surgical strategy for patients who develop iatrogenic flat back syndrome, especially after short-segment fusion surgery. This study aimed to evaluate the effectiveness of ACR in treating iatrogenic flatback syndrome following short-segment lumbar fusion and to identify preoperative radiographic parameters and their optimal cut-off values for determining the need for additional PSO.

## 2. Materials and Methods

This study was a retrospective analysis of patients with iatrogenic flatback syndrome who underwent surgery to reconstruct spinal alignment at our single institution, performed by two experienced spine surgeons. We reviewed 158 patients who developed iatrogenic flatback after previous short-segment fusion (≤3 levels) and underwent revision correction with ACR with or without PSO between March 2011 and May 2022. We excluded (1) previous long-segment fusion (≥ 4 levels); (2) underwent PSO without ACR or had a history of prior ACR, cervical, or lower extremity surgery; (3) patients with severe coronal imbalance (Cobb’s angle ≥ 40°); (4) patients with pathophysiological kyphosis, and (5) patients with improper or incomplete radiographs. Finally, we enrolled 49 patients who underwent deformity correction surgery for flatback syndrome that occurred after short-segment (≤3 levels) fusion surgery. These 49 patients were divided into two groups according to the surgical procedure: Group A (*n* = 33, only ACR) and Group B (*n* = 16, ACR combined with PSO). Among patients in Group A, an additional analysis was performed by dividing them into two subgroups: those who developed excessive distraction of anterior disc height during perioperative surgery (“Excessive group”) and those who did not (“Non-excessive group”).

### 2.1. Radiographic Evaluation

Preoperative and postoperative spinopelvic parameters were measured on standing lateral radiographs. These parameters included the sagittal vertical axis (SVA), thoracic kyphosis (TK), lumbar lordosis (LL), pelvic incidence (PI), pelvic tilt (PT), sacral slope (SS), lower lumbar lordosis (LLL), defined as the angle from the upper endplate of L4 to the upper endplate of S1, and Fused segment angle (FSA), which was defined as the angle between the superior endplate of the fused level and the inferior endplate of the fused level [14].

Flexion and extension lateral radiographs of the lumbar spine were used to assess lumbar flexibility and adjacent segment mobility. Dynamic lumbar lordosis (DL) was defined as the difference in the lumbar lordosis angle between flexion and extension. Dynamic segment angulation (DA) was defined as the change in adjacent segment angulation between flexion and extension [15].

### 2.2. Clinical Evaluation and Complications

**Figure 1:**
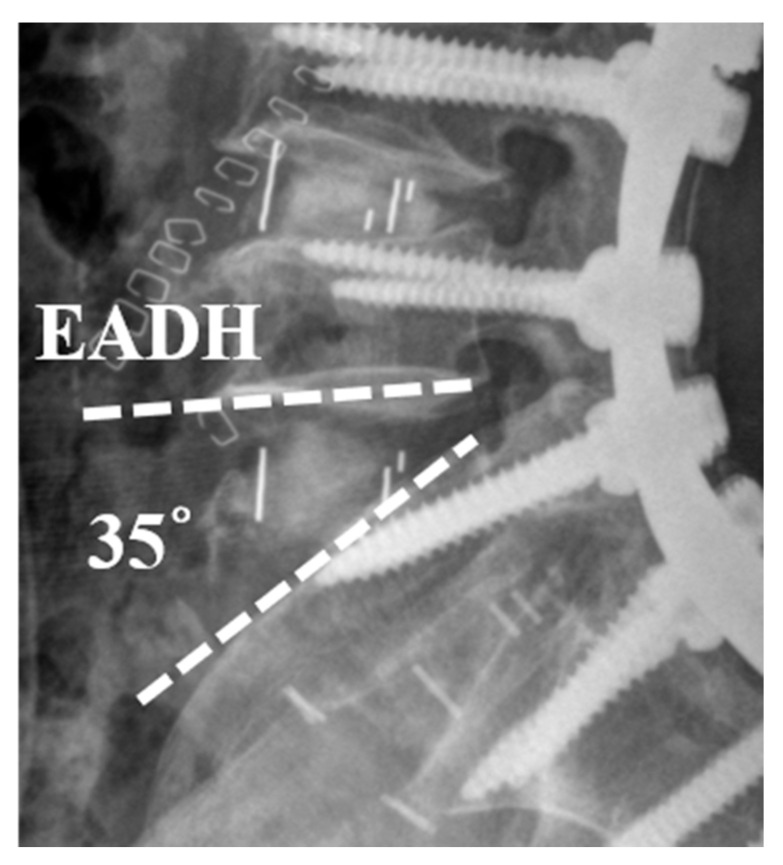
Excessive distraction of anterior disc height (EADH) was defined as the presence of an excessive anterior disc angle (>30°) between adjacent endplates.

Patient-reported outcome measures (PROMs) were assessed preoperatively and at 6 months postoperatively using the Oswestry Disability Index (ODI) and the Visual Analogue Scale (VAS) of low back. Perioperative major complications and postoperative complications were recorded. In terms of postoperative complications. Excessive distraction of anterior disc height (EADH) was defined as the presence of an excessive anterior disc angle (>30°) between adjacent endplates, measured on postoperative standing lateral radiographs (Figure 1).

### 2.3. Statistical Analysis

All statistical analyses were performed using SPSS (version 22, IBM SPSS Statistics). Student’s *t*-test was used to compare demographics, spinopelvic parameters, clinical outcomes, and complications between the two groups. Radiological spinopelvic parameters, postoperative complications, and patient-reported outcomes were compared between the two groups. Paired *t*-tests were used to compare preoperative and postoperative data within each group. A *p*-value < 0.05 was considered statistically significant. The Receiver Operating Characteristic (ROC) curve was used to determine the cut-off values for spinopelvic parameters associated with the decision to perform PSO.

## 3. Results

### 3.1. Demographic and Surgical Data

The patients enrolled in our study had an average age of 72.6 ± 6.6 in group A and 67.1 ± 6.8 in group B; both groups did not have significant differences (*p* = 0.420). In demographic and surgical data, in terms of body mass index (BMI), follow-up duration, instrumented level, or posterior lumbar interbody fusion (PLIF) segment, there was no significant difference (*p* > 0.05). Group A had fewer previous fusion levels than Group B (1.1 ± 0.2 vs. 2.2 ± 0.8, *p* < 0.001). The operation time in Group A was also significantly shorter than that in Group B (404.9 ± 91.4 min vs. 506 ± 154.8 min, *p* = 0.032). In contrast, the number of ACR levels did not have a statistically significant difference (2.5 ± 0.6 vs. 1.9 ± 0.9, *p*= 0.053); however, the estimated blood loss (EBL) in group B was slightly higher than in group A. Still, it did not have a statistically significant difference (1845 mL ± 929.9 vs. 2260 ± 1447.8 ml, *p* = 0.348). In Group B, the proportion of PSO level was located at L3 (*n* = 9, 56.3%) and L4 (*n* = 7, 43.7%) (Table 1).

### 3.2. Radiographic Parameters

When comparing radiographic parameters between the two groups (Table 2). Preoperatively, Group B showed a significantly greater C7-SVA compared to Group A (293.8 ± 90.3 mm vs. 219.8 ± 76.4 mm, *p* = 0.026). Although the postoperative C7-SVA did not differ significantly between groups (30.7 ± 45.5 mm vs. 61.7 ± 66.1 mm, *p* = 0.195), the correction was significantly larger in Group B (263.0 ± 79.9 mm vs. 157.9 ± 84.9 mm, *p* = 0.003). There were no statistically significant differences in TK, LL, LLL, T1PA, SS, PT, PI, or PI–LL mismatch in the two groups (*p* > 0.05). Notably, FSA showed significant differences between groups in both preoperative and postoperative parameters. Group B demonstrated a significantly less lordotic (more kyphotic) preoperative FSA (5.1 ± 8.0° vs. −7.2 ± 7.5°, *p* = 0.001), a more lordotic postoperative FSA (−26.6 ± 12.6° vs. −7.8 ± 7.8°, *p* < 0.001), and a significantly greater correction angle (−31.6 ± 8.9° vs. −0.6 ± 2.3°, *p* < 0.001) compared to Group A.

### 3.3. Clinical Outcomes and Complications

Both groups showed significant postoperative improvements in the VAS score of low back and ODI after 6 months (*p* < 0.05), and there were no significant differences between the groups preoperatively or postoperatively (*p* > 0.05) (Table 3).

The incidence of perioperative and postoperative complications is presented in Table 4. Perioperative motor deficit occurred in 1 patient in Group A and in 2 patients in Group B (6.1% vs. 12.5%, *p* = 0.440). All patients recovered within one year after the operation. Interestingly, seven patients (21.2%) in Group A experienced EADH perioperatively, whereas no patients in Group B did so (*p* = 0.047). There was no statistically significant difference in postoperative complications between the two groups, and no revision surgery was required.

### 3.4. Preoperative Radiographic Parameters Depend on Complication

Based on the EADH complication identified in Group A, we performed an additional analysis of preoperative spinopelvic parameters, lumbar flexibility, and adjacent segment motion between the Excessive and Non-excessive groups (Table 5). Regarding adjacent segment motion, DA was significantly smaller in the Excessive group than in the Non-excessive group (2.8 ± 1.1° vs. 9.1 ± 3.5°, *p* < 0.001). Preoperative LL in the Excessive group was 11.3 ± 13.6°, indicating a more kyphotic angle than the 4.1 ± 7.4° observed in the Non-excessive group, although this difference was not statistically significant (*p* = 0.054). Other preoperative radiographic parameters did not differ significantly between the two groups.

### 3.5. Receiver Operating Characteristic Curves Determining Optimal Cut-Off Values of Spinopelvic Parameters

**Figure 2:**
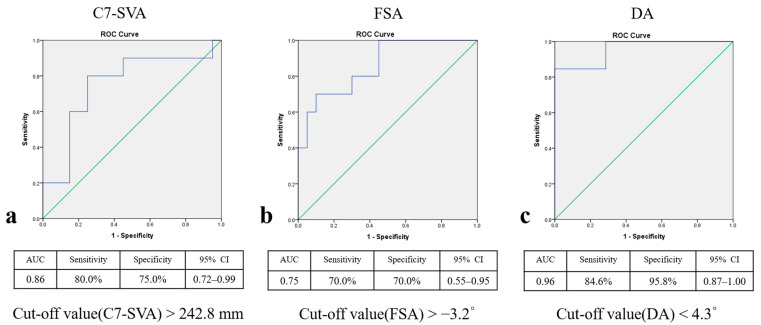
Receiver operating characteristic curves determining optimal cut-off values of spinopelvic parameters for indicating additional PSO. (**a**) C7 sagittal vertical axis (C7-SVA): cut-off value > 242.8 mm, AUC = 0.86; (**b**) Fused segment angle (FSA): cut-off value > −3.2°, AUC = 0.75; (**c**) Dynamic segment angulation (DA): cut-off value < 4.3°, AUC = 0.96. Sensitivity, specificity, and 95% CI are presented in each subfigure.

The optimal cut-off values of the preoperative radiographic parameters for selecting PSO were C7-SVA > 242.8 mm (95% confidence interval, 95% CI 0.72–0.99), FSA > −3.2° (95% CI 0.55–0.95), and DA < 4.3° (95% CI 0.87–1.0), respectively. The AUC was 0.86 for C7SVA, 0.75 for FSA, and 0.96 for DA. At these thresholds, the sensitivity and specificity for C7SVA were 80.0% and 75.0%, FSA 70.0% and 70.0%, while those for DA were 84.6% and 95.8% (Figure 2). However, these cut-offs are hypothesis-generating given the small sample and should be interpreted with caution pending external validation.

## 4. Discussion

**Figure 3:**
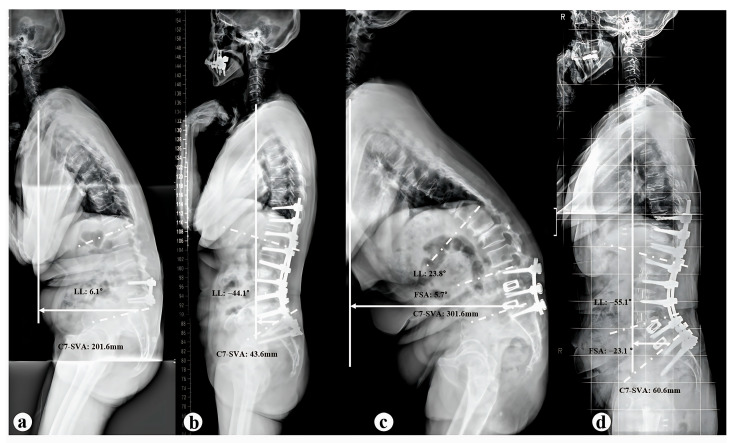
A 72-year-old female diagnosed with iatrogenic flatback following a prior spinal fusion from L4–5. (**a**) Preoperative radiographs show severe sagittal imbalance, with a C7-SVA of 201.6 mm and LL of 6.1°. (**b**) After ACR at L2–4 and PLIF at L5 to the sacrum, postoperative radiographs at 2-year follow-up show significant correction, with the C7-SVA reduced to 43.6 mm and LL improved to −44.1°. A 71-year-old female diagnosed with iatrogenic flatback following a prior spinal fusion from L4 to the sacrum. (**c**) Preoperative radiographs show severe sagittal imbalance, with a C7-SVA of 301.6 mm, LL of 23.8°, and FSA of 5.7°. (**d**) After ACR at L1–4 and an additional PSO at L3, postoperative radiographs at 2-year follow-up show significant correction, with the C7-SVA reduced to 60.6 mm, LL improved to −55.1°, and FSA corrected to −23.1°.

The surgical treatment of iatrogenic flatback is highly complex, as it involves revision surgery following a prior procedure. Given that the extent of correction plays a critical role in determining clinical outcomes (such as postoperative SVA and LL), meticulous preoperative surgical planning is essential. To date, ACR has been established as an effective technique for reconstructing spinal alignment in adult spinal deformity, offering a lower complication rate compared to pedicle subtraction osteotomy PSO [11,12]. This approach is particularly beneficial in cases of iatrogenic flatback, where previous posterior fusion surgery often results in significant scarring and adhesions around the dura and posterior elements [12]. These conditions necessitate complex dissections for adequate exposure, making PSO technically challenging at previously fused vertebral levels. Jason M. Frerich et al. [16] reported a case series demonstrating that performing ACR at the proximal segments in patients with iatrogenic flatback led to significantly improved radiographic spinopelvic and better clinical outcomes compared to those who underwent PSO. Similarly, in our study, most patients in group A had undergone prior short-segment fusion involving two or fewer levels. Despite presenting with notable preoperative deformities, they achieved satisfactory radiographic correction through ACR alone, particularly in key parameters such as C7–SVA, LL, and PI–LL mismatch (Figure 3a,b). These findings further support the effectiveness of ACR in restoring sagittal alignment in complex iatrogenic spinal deformities.

In our study, both groups showed severe preoperative C7-SVA, with Group A 219.8 ± 76.4 mm and Group B showing an even greater average of 293.8 ± 90.3 mm. These values are substantially higher than those reported in previous studies. For example, Munish et al. [7] analyzed 351 cases of iatrogenic flatback and reported an average SVA of 128.4 ± 70.6 mm, while Laine et al. [17] reported an average SVA of 121.6 ± 129.3 mm in a multicenter cohort of 273 patients with fixed sagittal imbalance. These earlier studies primarily focused on cases following long-level fusion, where the imbalance was mainly attributed to insufficient initial correction or subsequent loss of alignment over time. In contrast, our findings suggest that additional factors, such as progressive degenerative changes in the remaining flexible segments and the gradual decline in compensatory mechanisms, may contribute to the more pronounced sagittal malalignment observed in our cohort. These observations are consistent with those of Diebo et al. [10], who reported that segmental degenerative changes can induce compensatory alterations at adjacent levels, ultimately resulting in global sagittal imbalance. Moreover, as compensatory mechanisms deteriorate over time, particularly in elderly individuals, sagittal malalignment may become more severe despite relatively limited local deformity. It is further supported by the study by Cho et al. [18], which analyzed 34 elderly patients with degenerative sagittal imbalance and reported a mean SVA of 220.8 ± 78.3 mm. Their results highlighted that while degenerative imbalance may initially present as flexible, it tends to progressively stiffen, often leading to marked anterior deviation of the C7 plumb line exceeding 20 cm.

Although ACR is effective in many cases, it can be insufficient to achieve the angular correction required for proper deformity reconstruction in patients with severe sagittal imbalance or fixed sagittal deformity. In such settings, additional PSO may be necessary to optimize sagittal realignment. Prior studies have supported a hybrid ACR plus additional PSO strategy for severe, fixed deformity—reporting greater restoration of segmental lordosis and PI–LL as well as potential biomechanical advantages, including reduced risks of rod fracture and hardware failure [19,20]. However, these reports have not clearly delineated the specific preoperative indications for performing additional PSO. Accordingly, we aimed to identify which preoperative characteristics are associated with the need for additional PSO.

In the current study, when the previous fusion segment extended beyond two levels and was associated with a more severe global malalignment, we frequently performed an additional PSO. This approach was necessary due to the limited number of flexible segments available for ACR to sufficiently correct such severe spinal deformities. Although postoperatively, the number of ACR levels between the two groups did not show a significant difference. This was because, in Group B, ACR was frequently extended to the L1–L2 level, whereas in Group A, it was not. Performing ACR at this level presents technical challenges due to the subdiaphragmatic location of L1–L2, which is often obstructed by the 12th rib and diaphragm. These anatomical constraints can limit surgical exposure and hinder the placement of hyperlordotic cages [11]. Consequently, achieving sufficient segmental lordosis at L1–L2 was often difficult, necessitating the use of additional PSO to accomplish appropriate sagittal realignment [16]. As a result, Group B achieved a significantly greater correction in C7-SVA compared to Group A (*p* = 0.003, Table 2). Further analysis using ROC curve identified a preoperative C7–SVA cut-off value of 242.8 mm as a significant predictor for the necessity of additional PSO (Figure 2a). This finding suggests that patients with sagittal imbalance exceeding this threshold are unlikely to achieve sufficient realignment through ACR alone and may require more extensive corrective strategies, such as the addition of PSO, to effectively restore global sagittal alignment.

In addition to severe sagittal imbalance, PSO is also indicated for kyphotic spinal deformities, such as post-traumatic kyphosis. Wenhao Hu et al. [21], reported that PSO combined with interbody cage insertion resulted in favorable outcomes in patients with post-traumatic kyphosis. A substantial correction angle was achieved safely, as the procedure aids in restoring spinal column height and reducing the risk of spinal cord buckling or kinking. Despite short-segment fusion surgery, focal iatrogenic kyphosis remains a potential complication, often resulting from technical factors such as suboptimal cage positioning, inadequate rod contouring, or interbody cage subsidence. As reported by Diebo et al. [10], insufficient attention to segmental alignment and device placement may inadvertently induce kyphosis, particularly in levels with preserved disc height and preexisting lordosis. In such cases, PSO may be necessary as an additional corrective measure to restore sagittal alignment. In our cohort, patients in Group B who underwent additional PSO showed a significantly more kyphotic FSA compared to those in Group A who did not (*p* = 0.001), suggesting that segmental hypolordosis or kyphosis may serve as a key radiographic indicator for the necessity of PSO (Figure 3c,d). Further ROC curve analysis identified a preoperative FSA cut-off value of less than 3.2° as predictive of the need for additional PSO (Figure 2b). These findings suggest that PSO should be considered when the fused segment fails to achieve or maintain appropriate lordotic alignment, particularly in cases with residual or progressive segmental kyphosis. The primary PSO vertebrae were L3 (56.3%) and L4 (43.7%), which is consistent with prior research. G. Lainé et al. [17], reported in a multicenter cohort study that PSO levels were predominantly at L3 and L4 (75.3%) in patients with fixed sagittal imbalance. Similarly, Munish C. Gupta et al. [7], found that L3 (43.6%) was the most common PSO site in revision patients. To our knowledge, PSO should be performed at the level of maximal deformity to achieve optimal sagittal correction [22]. In patients with iatrogenic flatback, prior fusion was frequently located at the lower lumbar spine (L4 to S1), and due to adjacent segment degeneration or focal kyphosis within the fused segment, the upper instrumented vertebra or the immediately adjacent vertebra often becomes the apex of the deformity [16,22]. Additionally, performing PSO above L3 poses an increased risk of neurological deficits due to the proximity of the spinal cord and conus medullaris [23]. This risk is particularly concerning in patients with prior posterior surgery, where the posterior elements may be extensively scarred and adhered, making surgical exposure and correction more complex [12].

There was no significant difference in ODI and VAS for low back pain between the two groups, either preoperatively or postoperatively. However, both groups showed significant postoperative improvement (*p* < 0.05). Although the surgical methods differed between the two groups, as shown in Table 2, there was no difference in postoperative spinopelvic parameters. This may explain why there was no significant difference in clinical outcomes between the groups after surgery. Nevertheless, previous studies [22,24,25] have suggested that achieving a C7-SVA less than 5 cm and a PI-LL mismatch less than 10° is associated with improved quality of life and functional outcomes. However, in elderly patients (age > 70 years), several studies [3,22] recommend achieving less stringent goals, specifically a C7-SVA less than 8 cm and a PI-LL mismatch less than 15°. These recommendations align closely with our study results.

Interestingly, seven cases of EADH were observed exclusively in patients who underwent ACR alone, despite the use of the same hyperlordotic cage. This phenomenon may be attributed to increased mechanical stress at adjacent segments with pre-existing degeneration, particularly involving the anterior longitudinal ligament (ALL). Minor intraoperative trauma, such as inadvertent injury to the ALL during disc preparation or anterior distraction during cage insertion and rod application, may predispose these segments to localized structural failure. To enhance segmental stability, supplemental dual rods were applied at the adjacent level during the perioperative period. However, two of these cases progressed to nonunion by two years postoperatively and required revision surgery. As a result, the anterior disc height may exceed the intended correction angle of the hyperlordotic cage, which is typically 23° to 30° [13]. This hypothesis is consistent with the rationale for performing ACR at flexible and non-ankylosed segments, where controlled correction is more safely achievable [11]. It also implies that only ACR alone cannot achieve an appropriate lordotic curve, so posteriorly based spinal osteotomy, such as PSO, is required. Therefore, we subdivided group A into two subgroups to investigate the optimal timing for performing additional PSO. There were no significant differences in preoperative spinopelvic parameters between the two subgroups. Although DL also did not significantly differ, DA in the excessive subgroup was 2.8 ± 1.1°, significantly lower than that in the Non-excessive group (9.1 ± 3.5°, *p* = 0.001). These findings suggest that the flexibility of the adjacent segment plays a more crucial role in deciding to perform additional PSO than the overall lumbar flexibility. In our cohort, ROC curve analysis suggested an association between DA < 4.3° and both EADH and the selection of additional PSO; given the small sample size, this observation is hypothesis-generating and should be validated externally before routine application (Figure 2c).

The primary limitation of this study is the small sample size and its single-center, retrospective design; despite strict inclusion and exclusion criteria focusing exclusively on patients with prior short-segment fusion who subsequently developed iatrogenic flatback deformity, the cohort was constrained because cases involving limited revisions of one or two adjacent levels for adjacent segment disease were excluded, even as the incidence of iatrogenic flatback has risen in recent years. In addition, the nonrandomized, single-center, revision-only design introduces potential selection bias and confounding by indication and surgeon preference, and learning-curve effects may have influenced procedure selection and perioperative management, which together limit generalizability. Patient-reported outcomes were available only in the short term, and ODI/VAS did not differ between groups, cautioning against inferring symptomatic benefit solely from radiographic correction. Finally, the preoperative values proposed to guide consideration of additional PSO were derived from a modest dataset; ROC-based values should be regarded as exploratory, with a risk of overfitting, and require external validation. Prospective, multicenter studies with standardized indications, longer follow-up, and external validation are needed to confirm these observations.

## 5. Conclusions

ACR alone and ACR combined with PSO showed satisfactory radiographic outcomes in patients with iatrogenic flatback after short-segment fusion. For selected patients with preoperative C7SVA > 242.8 mm, FSA > −3.2°, or DA < 4.3°, additional PSO may be reasonable to help optimize sagittal alignment, particularly when segmental flexibility is limited or greater angular correction is required. However, these values are exploratory and should inform rather than determine surgical planning; external validation, ideally in multicenter cohorts, is warranted.

## Figures and Tables

**Table 1:** Demographic and Surgical Data.

Variables	Group A (*n* = 33)	Group B (*n* = 16)	*p* Value
Age, years	72.6 ± 6.6	67.1 ± 6.8	0.420
Sex (Male:Female)	3:30	1:15	0.733
EBL (mL)	1845 ± 929.9	2260 ± 1447.8	0.348
BMI (%)	25.2 ± 3.2	25.5 ± 2.2	0.742
Operative time, minutes	404.9 ± 91.4	506.0 ± 154.8	0.032 *
Follow-up, moth	25.2 ± 5.8	25.2 ± 4.1	0.981
No. of previous fusion level	1.1 ± 0.2	2.2 ± 0.8	<0.001 *
No. of instrumented level	7.6 ± 0.8	8.1 ± 0.3	0.530
No. of ACR level	2.5 ± 0.6	1.9 ± 0.9	0.053
PLIF L5-S1, no. (%)	16 (40.8)	4 (25)	0.117
PSO, no. (%)			
L3		9 (56.3)	
L4		7 (43.7)	

Values are presented as mean ± SD unless otherwise indicated. * Significant difference. *n* = number of patients; EBL: Estimated blood loss; BMI: Body mass index; ACR: Anterior column realignment; PSO: Pedicle subtraction osteotomy.

**Table 2:** Comparison of Radiographic Parameters Between Group A and Group B.

Parameter	Group A (*n* = 33)	Group B (*n* = 16)	*p* Value
C7-SVA (mm)			
Preoperative	219.8 ± 76.4 †	293.8 ± 90.3 †	0.026 *
Postoperative	61.7 ± 66.1 †	30.7 ± 45.5 †	0.195
correction	157.9 ± 84.9	263.0 ± 79.9	0.003 *
TK (°)			
Preoperative	8.2 ± 14.1 †	−0.4 ± 12.2 †	0.110
Postoperative	33.4 ± 12.6 †	29.0 ± 13.5 †	0.389
correction	−25.2 ± 13.1	−29.5 ± 18.8	0.372
LL (°)			
Preoperative	9.6 ± 19.4 †	22.2 ± 20.5 †	0.051
Postoperative	−51.1 ± 10.2 †	−49.8 ± 8.7 †	0.728
correction	60.7 ± 23.3	72.0 ± 17.8	0.100
LLL (°)			
Preoperative	−7.0 ± 9.8 †	−2.4 ± 13.3 †	0.287
Postoperative	−23.9 ± 8.9 †	−27.6 ± 10.9 †	0.331
correction	16.9 ± 10.4	25.2 ± 5.8	0.127
T1PA (°)			
Preoperative	53.5 ± 15.4 †	64.6 ± 17.3 †	0.085
Postoperative	20.8 ± 11.1 †	21.8 ± 8.9 †	0.820
correction	32.7 ± 16.3	42.8 ± 12.9	0.099
SS (°)			
Preoperative	24.4 ± 10.8 †	26.4 ± 9.1 †	0.621
Postoperative	38.1 ± 7.0 †	34.3 ± 9.6 †	0.230
correction	−13.6 ± 8.4	−7.9 ± 9.1	0.097
PT (°)			
Preoperative	34.1 ± 12.1 †	33.4 ± 9.3 †	0.885
Postoperative	20.1 ± 9.5 †	25.1 ± 10.6 †	0.212
correction	13.9 ± 8.6	8.4 ± 9.0	0.114
PI (°)			
Preoperative	58.5 ± 11.0	59.9 ± 5.2	0.705
Postoperative	58.3 ± 11.0	59.6 ± 5.4	0.738
correction	0.1 ± 1.2	0.2 ± 1.2	0.726
PI-LL (°)			
Preoperative	65.1 ± 19.1 †	82.1 ± 20.9 †	0.051
Postoperative	7.2 ± 6.2 †	9.8 ± 11.1 †	0.658
correction	57.8 ± 23.4	72.2 ± 17.4	0.096
FSA (°)			
Preoperative	−7.2 ± 7.5	5.1 ± 8.0 †	0.001 *
Postoperative	−7.8 ± 7.8	−26.6 ± 12.6 †	<0.001
correction	−0.6 ± 2.3	−31.6 ± 8.9	<0.001

Values are presented as mean ± SD unless otherwise indicated. * Significant difference between two groups (*p* < 0.05). † Significant difference between preoperative and postoperative (*p* < 0.05).TK: Thoracic kyphosis; LL: Lumbar lordosis; LLL: Lower lumbar lordosis; T1PA: T1 pelvic angle; SS: Sacral slope; PT: Pelvic tilt; PI: Pelvic incidence; PI-LL: Pelvic incidence minus lumbar lordosis; FSA: Fused segment angle.

**Table 3:** Comparison of Clinical Outcomes.

Variables	Group A (*n* = 33)	Group B (*n* = 16)	*p* Value
ODI			
Preoperative	28.1 ± 7.8	23.5 ± 7.6	0.146
Postoperative	14.5 ± 7.4	11.0 ± 7.5	0.229
*p* Value	<0.001 *	0.001 *	-
VAS-low back			
Preoperative	5.7 ± 2.1	5.4 ± 2.2	0.724
Postoperative	2.9 ± 1.3	2.6 ± 1.3	0.614
*p* Value	<0.001 *	0.009 *	-

Values are presented as mean ± SD unless otherwise indicated. * Significant improvement (*p* < 0.05) ODI: Oswestry Disability Index; VAS-low back; Visual Analogue Scale of low back.

**Table 4:** Comparison of incidence of complication between two groups.

	Group A (*n* = 33)	Group B (*n* = 16)	*p* Value
Perioperative *n*, (%)			
Dura tear	2, (6.1%)	2, (12.5%)	0.440
Motor deficit	1, (3.0%)	2, (12.5%)	0.195
Blood loss (>4000 mL)	0	1, (6.3%)	0.147
EADH	7, (21.2%)	0	0.047 *
Postoperative *n*, (%)			
Infection	1, (3.0%)	1, (6.3%)	0.593
PJK	2, (6.1%)	1, (6.3%)	0.979
PJK	2, (6.1%)	1, (6.3%)	0.979
Rod fracture	3, (9.1%)	2, (12.5%)	0.712

* Significant difference between two groups (*p* < 0.05). EADH: Excessive distraction of anterior disc height.PJK: Proximal junctional kyphosis

**Table 5:** Preoperative Radiographic Parameters depend on complications.

Parameter	Excessive Group (*n* = 7)	Non-Excessive Group (*n* = 26)	*p* Value
C7-SVA (mm)	213.2 ± 83.9	223.3 ± 75.4	0.785
TK (°)	12.2 ± 10.5	7.2 ± 6.5	0.081
LL (°)	11.3 ± 13.6	4.1 ± 7.4	0.054
LLL (°)	−5.6 ± 12.7	−7.8 ± 8.3	0.643
PT (°)	36.4 ± 9.9	32.8 ± 13.4	0.544
PI (°)	56.2 ± 9.2	59.7 ± 12.1	0.509
DL (°)	18.6 ± 10.5	21.7 ± 7.4	0.458
DA (°)	2.8 ± 1.1	9.1 ± 3.5	<0.001 *

Values are presented as mean ± SD unless otherwise indicated. * Significant difference between two groups (*p* < 0.05). DL: Dynamic lumbar lordosis; DA: Dynamic segmental angle.

## Data Availability

The datasets used and analysed during the current study are available from the corresponding author on reasonable request.

## Abbreviations

The following abbreviations are used in this manuscript:

ACR	Anterior column realignment
ALL	Anterior longitudinal ligament
BMI	Body mass index
C7SVA	C7 sagittal vertical axis
DA	Dynamic segment angulation
DL	Dynamic lumbar lordosis
DSI	Degenerative sagittal imbalance
EADH	Excessive distraction of anterior disc height
EBL	Estimated blood loss
FSA	Fused segment angle
LL	Lumbar lordosis
LLL	Lower lumbar lordosis
ODI	Oswestry Disability Index
PI	Pelvic incidence
PI–LL	Pelvic incidence minus lumbar lordosis
PLIF	Posterior lumbar interbody fusion
PROMs	Patient-reported outcome measures
PSO	Pedicle subtraction osteotomy
PT	Pelvic tilt
ROC	Receiver operating characteristic
SVA	Sagittal vertical axis
SS	Sacral slope
TK	Thoracic kyphosis
